# Metallic Biomaterials: Current Challenges and Opportunities

**DOI:** 10.3390/ma10080884

**Published:** 2017-07-31

**Authors:** Karthika Prasad, Olha Bazaka, Ming Chua, Madison Rochford, Liam Fedrick, Jordan Spoor, Richard Symes, Marcus Tieppo, Cameron Collins, Alex Cao, David Markwell, Kostya (Ken) Ostrikov, Kateryna Bazaka

**Affiliations:** 1 School of Chemistry, Physics and Mechanical Engineering, Queensland University of Technology, Brisbane, QLD 4000, Australia; me.chua@connect.qut.edu.au (M.C.); madison.rochford@connect.qut.edu.au (M.R.); liam.fedrick@connect.qut.edu.au (L.F.); jordan.spoor@connect.qut.edu.au (J.S.); richard.symes@connect.qut.edu.au (R.S.); marcus.tieppo@connect.qut.edu.au (M.T.); ck.collins@connect.qut.edu.au (C.C.); alex.cao@connect.qut.edu.au (A.C.); david.markwell@connect.qut.edu.au (D.M.); Kostya.ostrikov@qut.edu.au (K.O.); 2 CSIRO-QUT Joint Sustainable Processes and Devices Laboratory, Commonwealth Scientific and Industrial Research Organization, P.O. Box 218, Lindfield, NSW 2070, Australia; 3 Institute of Health and Biomedical Innovation, Queensland University of Technology, Brisbane, QLD 4000, Australia; 4 College of Science and Engineering, Technology and Engineering, James Cook University, Townsville, QLD 4810, Australia; olga.bazaka@jcu.edu.au

**Keywords:** biomaterial, inflammation, implant, advanced materials, surface modification

## Abstract

Metallic biomaterials are engineered systems designed to provide internal support to biological tissues and they are being used largely in joint replacements, dental implants, orthopaedic fixations and stents. Higher biomaterial usage is associated with an increased incidence of implant-related complications due to poor implant integration, inflammation, mechanical instability, necrosis and infections, and associated prolonged patient care, pain and loss of function. In this review, we will briefly explore major representatives of metallic biomaterials along with the key existing and emerging strategies for surface and bulk modification used to improve biointegration, mechanical strength and flexibility of biometals, and discuss their compatibility with the concept of 3D printing.

## 1. Introduction

The use of implants has grown dramatically over the past years, driven by ageing of populations in developed countries, and the desire of the patients to maintain the same level of activity and quality of life. Consequently, the demand for high-performance implantable biomaterials that can address unique challenges in cardiology, vascular therapy, orthopaedics, trauma, spine, dental and wound care has also been increasing steadily. Indeed, the biomaterial market was valued at $94.1 billion USD in 2012 and is currently worth $134.3 billion USD in 2017 [1]. The diversity and functionality of available biomaterials, as well as the methods for their processing and assembly into an implantable device, have also experienced substantial growth, with a wide variety of synthetic, natural and hybrid materials currently on the market [2,3,4,5]. Such diversity allows for better selection of the material to meet the specific objectives of the treatment, such as using metals with have high electro conductivity as electrodes in artificial organs, chemically inert materials for permanent replacement of lost function, or biodegradable materials as a temporary framework for cases where regeneration of lost tissue or function is possible [6,7].

**Figure 1:**
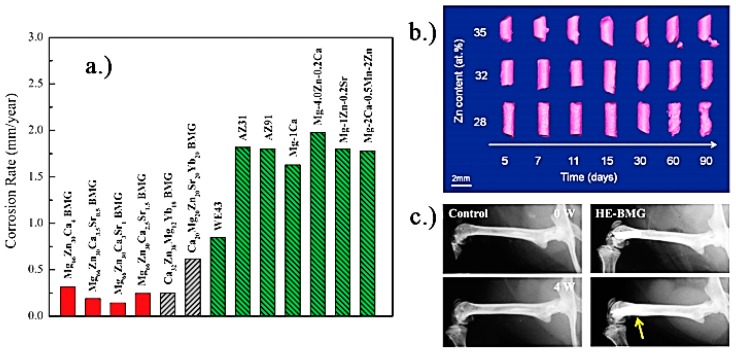
(**a**) Corrosion rates of Mg-based alloys in physiologically relevant solutions [12]; (**b**) Images of Mg95–xZnxCa5 (at %) implanted in rat femurs, reconstructed from in vivo μ-CT scans. Note visible degradation after 30 days for the x = 28 sample, while minimal and no degradation are visible on the x = 32 and x = 35 samples, respectively [13]; (**c**) Radiographs of mice distal femora with and without implanted high-entropy CaMgZnSrYb alloy, immediately after implantation, and 4 weeks postoperatively. The sample shows no gas formation, no inflammation, and enhanced circumferential osteogenesis in the implanted bone (yellow arrow), indicating new bone formation [14]. Reproduced with permission from [14,15].

Importantly, recently there has been a significant emphasis on multi-functionality of chosen biomaterials. For instance, a temporary scaffold material may not only provide physical support for tissue regeneration, but may also be loaded with biological factors, such as bone morphogenetic protein-2 (BMP-2), transforming growth factor-*β* (TGF-*β*), fibroblast, platelet-derived and vascular endothelial growth factors (FGF, PDGF, VEGF) and others, to stimulate cell attachment and tissue formation and/or chemotherapy agents, to selectively target cancer cells not removed during surgery, or release desirable molecules and ions during scaffold biodegradation. This multi-functionality is well-illustrated by magnesium implants. Magnesium has sufficient tensile strength, resistance to fracture [8,9], and light weight to support such load-bearing applications as stenting or small fracture repair [10], and as it degrades, it releases Mg ions which are essential for human metabolism and are known to provide stimulatory effects on the generation of new bone tissue [11]. The biodegradation kinetics of the Mg scaffold can be controlled by the nature of the alloying metals (as shown in Figure 1), as well as certain forms of mechanical processing and coating.

Imparting multi-functionality on bio-inert metals, such as Ti- and Co- based alloys, is generally achieved by surface modification, such as surface structuring or coating with bioactive ceramic and polymer thin films. Bio-inert materials, most commonly based on Ti, Co, and steel, are critical for many load-bearing functions, where their resistance to corrosion provides excellent long-term stability and reliable mechanical strength, with minimal long-term toxicity to the host locally or on systemic level [16,17]. These materials have excellent tensile strength, fracture toughness and fatigue stress [18,19], and over the years, they have found applications in orthopaedics as artificial joints, plates and screws, orthodontics as braces and dental implants, cardiovascular and neurosurgical devices such as components of artificial hearts, staples, stents and wires. Among bio-inert materials, titanium is often the material of choice due to a favourable combination of biocompatibility, corrosion resistance, strength and elastic modulus, and relatively low weight and density when compared to conventional steel and Co-Cr alloys [20]. 

There are several important challenges associated with the use of implantable metals. For instance, the control over biodegradation kinetics is critical for resorbable metals, such as Mg or Fe, where untimely degradation may lead to premature loss of mechanical strength before the tissue or function has been fully restored. On the other hand, long-term presence of metals such as steel, Co-Cr or Ti alloys in the body is associated with an increased risk of development of cutaneous and systemic hypersensitivity reactions, whereas a relatively high modulus of these metals compared to natural bone tissue leads to stress shielding and consequent osteopenia [17]. Characteristic of any implant is the risk of infection and inflammation, which can significantly undermine the performance of an implant [21,22] and lead to a significant loss of tissue in the proximity of the implant [23]. For load-bearing implants, septic or aseptic loosening of the implant may interfere with the transfer of forces, such as the incorrect transfer of the biting force to the dental implant and the surrounding bone, leading to mechanical failure once the fatigue strength of the dental implant is reached [24]. Considering that peri-implantitis was reported in 20% of patients 8–10 years after the implant placement [25], such complications come at a significant cost to the healthcare and to the health and well-being of the patient [26,27].

Not surprisingly, considerable effort has gone into addressing these issues, with a wealth of surface and bulk modification techniques developed for a wide range of materials, including laser ablation, plasma and acid etching, surface functionalisation, coating, ion implantation, grain refinement to name but a few. In addition to the chemical structure of the material, the manner in which it is formed into an implant is undergoing significant development [28]. Recently, three-dimensionally printing of biomaterials has emerged as a technique that can enable fabrication of complex structures ideally matched to the needs of the individual patient [28,29,30]. Importantly, in addition to matching the macroscopic size and anatomical shape of the implant to that of the lost tissue, the method of construction used in 3D printing may enable replication of the nano- and micro-scale features within the bulk of the material. As mentioned earlier, bio-inert implants, such as those fabricated from smooth, solid Ti and Co-Cr alloys, are often associated with limited osseointegration and stress shielding-induced osteopenia. Imparting size- and distribution-controlled porosity during computer-aided layered assembly may not only create a structure that better resembles extracellular matrix of healthy tissues and thus is more amenable to osseointgration, but also notably reduce the elastic modulus of biometals and thus minimise stress shielding.

Such an approach to impart multi-functionality on metallic implants is clearly highly attractive, not least because of the fact that unlike other methods, where several parts are individually-processed and then assembled, 3D bioprinting promises to produce the final complex product in a single process, significantly reducing the time and cost of manufacturing. However, 3D printing is ridden with challenges, particularly when it comes to processing of metals where melting or the use of nanoparticle is necessary to enable assembly. Furthermore, it is not clear to which extent the gains made from other methods of pre- or post-processing will be retained in the final structure should it be processed with 3D printing. 

In this review, we will briefly explore major representatives of metallic biomaterials along with the key existing and emerging strategies for surface and bulk modification used to improve biointegration, mechanical strength and flexibility of biometals, and discuss their compatibility with the concept of 3D printing. 

## 2. 3D Printing

The 3D printing technology has undergone significant development, and is currently used in a wide range of fields, from fashion to architecture and aircraft engineering. In medical and healthcare fields, 3D printing promises to address the needs of personalised medicine, producing solutions that match the individual anatomical needs of patients, and to advance tissue and organ engineering through printing of cells and complex multi-material scaffolds for tissue regeneration [31]. The former application has been utilised clinically in the production of personalised orthopaedics and stomatology solutions, whereas the latter at this stage is mostly limited to the printing of 3D tissue scaffolds under laboratory conditions. Clearly, the ability to produce implants that provide a good match to the anatomy of the patient is a valuable attribute of 3D printing, particularly for reconstructive surgery to treat craniofacial breaks or fractures [32], for patients suffering from dwarfism, or in cancer patients, where the implant is made to match the excised tissue and thus may reduce the pressure placed onto the existing bone compared with a non-customised implant [33]. Yet, it is a combination of these two approaches that hold most promise for the future of the personalised medicine.

Indeed, 3D printing relies on the use of computer-aided design and modelling to process the high-quality 3D image data of the anatomical structure acquired from the patient using computer tomography (CT), and to produce a model that reflects the anatomic surfaces and corrects for any defects. The model is then used to generate data for rapid prototyping of the implant with high dimensional accuracy. In addition to prescribing the macroscopic features of the implant, the model can be used to impart desirable structure to the bulk of the material, such as adding porosity where the shape, size, orientation and pore connectivity can be controlled to facilitate tissue in-growth, vessel formation and supply of nutrition to support the newly developed tissues [34]. Importantly, in principle, multiple materials can be printed at the same time, producing complex structures resembling that of tissues and organs in a single process [35]. Metals, natural and synthetic polymers, glasses, ceramics, active molecules, such as proteins and factors, and living cells can be integrated into a single structure with precision and accuracy [34]. 

Yet, there are a number of technological challenges that hinder the advancement of 3D printing. From the biological standpoint, the most significant challenge in 3D printing is the integration of a vascular network, without which the engineered 3D tissue or organ will not receive sufficient supply of nutrients, and necessary level of gas exchange and waste removal, all of which are essential for maturation during perfusion [36] and would directly affect cell viability and artificial organ performance. Yet, design and fabrication of a system which allows for the efficient transport of nutrients, growth factors and oxygen to the cells without interfering with the actual metabolic pathways remains a challenge. Indeed, despite the fact that several researchers have been trying to develop vascular trees using computer models [37], only a few attempts have been made towards making branched channels and so useful mechanically integrated bifurcated vessels are yet to be available [34,36].

A considerable technological challenge is the selection of biomaterials to be printed, since they should present a desirable combination of material and mechanical properties that make them amenable to processing and assembly with the necessary degree of accuracy and fidelity, and, once assembled, suitable for cell infiltration and attachment, and tissue formation [38]. The ambition of 3D printing is the formation of stable architectures with controlled nano-, micro-, and macro-scaled features superior to that attainable with such techniques as gas foaming, solvent casting with particle leaching, freeze-drying and electrospinning, and in particular, incorporation of complex internal architectures and curved channels, and gradients of features, where 3D printing is expected to excel over conventional techniques [39,40]. Yet, in practice, this is not a trivial matter, since pores may collapse or close during assembly, affecting structural organization and functionality. In inkjet printing, this can take place when liquid ink solidifies into a solid structure [28]. To maintain accuracy, it may be necessary to slow down the process so to enable complete solidification of the deposit prior to application of the next layer. Another issue is the formation of surface chemical or physical features that may not be compatible with cell attachment and proliferation, affecting the viability and performance of the system [41]. Achieving biologically-relevant cell densities with inkjet printing is also challenging, since higher concentrations of cells may impede correct droplet formation, lead to nozzle clogging and increase shear stress [42,43].

Economic costs of 3D printed parts also need to be considered. At present, the cost of printing of personalized surgical tools and implants is significantly higher than that of mass-produced alternatives, with further costs associated with the additional scans necessary to gather that data. On the other hand, the use of customized surgical items have been shown to notably reduce surgical time, and enhance the medical outcome of the surgery, thus potentially reducing the duration of the hospital stay and minimize the risk of revision surgeries [44]. Furthermore, the use of 3D parts is associated with reduced radiation exposure, which may potentially reduce the radiation burden and decrease the likelihood of developing radiation-associated side-effects [44].

For printing of metals and their alloys, the most common techniques include drop-on-powder deposition methods, such as selective laser sintering (SLS) of metal powders, direct metal laser sintering (DMLS) of alloy metal powders, electron beam melting (EBM) for titanium alloy powders, and continuous deposition methods, such as fused deposition modelling (FDM) of eutectic metals. Each method offers a unique set of advantages and challenges. For example, SLS uses a CO_2_ laser beam to produce implants with relatively high fidelity (with maximum standard errors of 0.1−0.6 mm), yet takes relatively long time (of ~15 h), and requires post-deposition sandblasting to finish the surface [45]. Typically used for layer-by-layer assembly of thermoplastics, such as acrylonitrile butadiene styrene, FDM can be used for deposition of eutectic alloys due to their relatively low melting temperature. Since a period of time is necessary for each subsequent layer to harden and bond to the underlying structure, a foam-based support is typically necessary. Inject printing in combination with powder-bed technology uses a low-viscosity liquid adhesive to bind adjacent powder particles in a 2D pattern for layer-by-layer assembly, however the resolution of such structures is relatively poor due to spreading of the liquid and so is the mechanical strength. The resolution of the printed structure is also affected by the wettability of the metal particles, where excessively low or high wetting of fine powder particles may lead to powder bed rearrangement or reduce the smallest feature size, respectively [46]. The mechanical strength of these constructs is dependent on the size of the particles, since the latter affects pore size distribution within the powder bed, and consequently, drop penetration [46].

Significantly better resolution can be attained by using advanced inkjet printing, such as the electro-hydrodynamic inkjet printing (e-jetP), where an electric field is used to overcome the effect of surface tension and eject the droplet of the solution containing metallic nanoparticles, e.g., Ag, Cu, Au or Co. The solvent is designed to evaporate quickly so that the layer-by-layer assembly is fast, with good control over the shape, dimension, and resolution of nano- and micro-scale features [47]. Thus-fabricated structures may be well suited as implantable electrodes and sensors, yet the utility of this technique for fabrication of macroscopic implants is presently limited. 

## 3. Permanent Metallic Implants

Among bio-inert metals, surgical stainless steel (316L), cobalt-chromium (CoCr) alloys and titanium (Ti) alloys are the most commonly used metals for fracture fixation, angioplasty and bone remodelling [18]. This is primarily due to their long-term stability under highly-reactive in vivo conditions and excellent mechanical properties [18]. It is important to note that while these materials are considered to have low corrosion, wear, friction, and highly-aggressive microenvironment may lead to material degradation, and associated release of unwanted metallic ions, which may induce local tissue damage and inflammatory reactions, such as gradual osteolysis of adjacent tissues, as well as systemic damage, such as metal hypersensitivity. The osteolysis may undermine the fixation and ultimately the loading and force transfer of the implant, leading to implant failure, corrective surgeries or post-surgery complications [48]. 

### 3.1. Stainless Steel

Before the introduction of stainless steel in the biomedical industry, implants were fabricated from pure metals, which often displayed lower corrosion resistance and mechanical strength [49]. These limitations were addressed to some extent with the introduction of 18/8 stainless steel in 1920s by W.H. Hatfield [50]. The 18/8 stainless steel showed better corrosion resistance, resulting in better long-term medical outcomes and less post-surgery complications. The incidence of implant failure and metal sensitivity was also considerably lower, with the latter being an important issue which may undermine the possibility of future use of other metals in the host. The high corrosion resistance of stainless steel stems from its possession of a high chromium content (>12 wt %) along with nickel and molybdenum, whereas carbon content was minimised to limit the formation of chromium carbides during hot work treatments, and thus maintain high chromium content [51].

**Figure 2:**
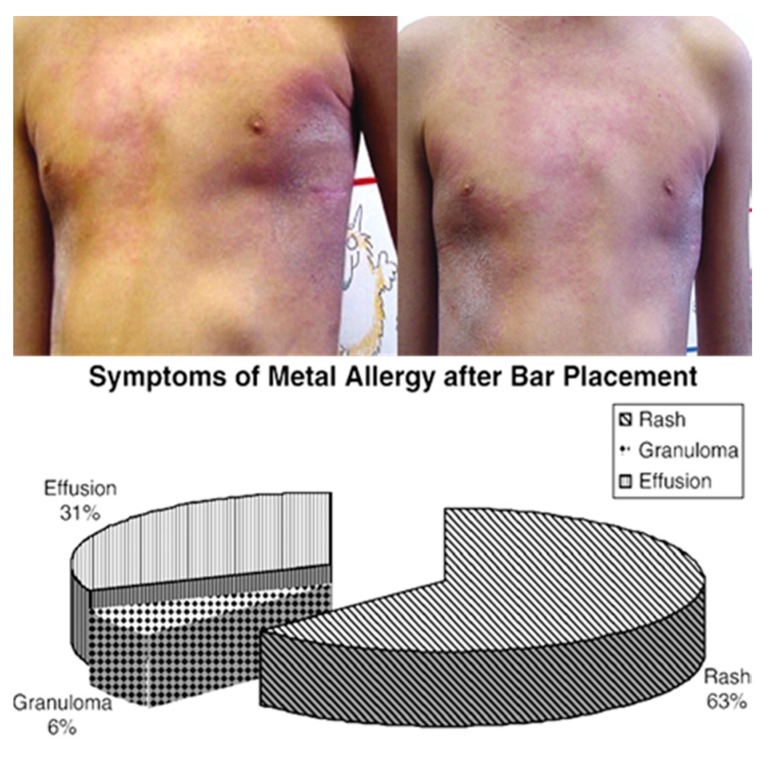
Top image: Photographs of a patient with erythema and rash after implantation of a stainless steel bar. Bottom image: typical symptoms reported in patients implanted with steel bars as part of a Nuss procedure for repair of pectus excavatum. Patients did not have a history of metal allergy prior to implantation. Reproduced with permission from [52].

**Figure 3:**
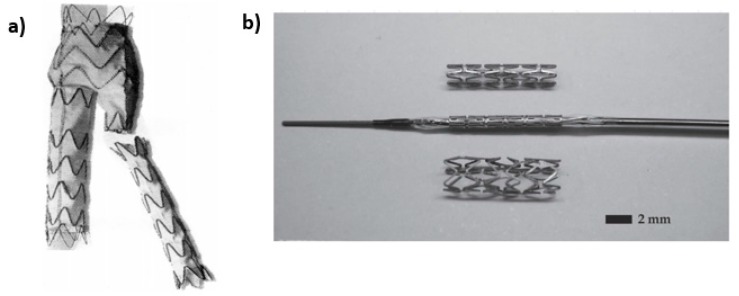
(**a**) Stent to be inserted in artery to keep the passageway open. Commonly made of stainless steel; (**b**) Prototype of biodegradable stent; (top) as fabricated; (mid) crimped onto a balloon catheter, and (bottom) expanded to 3 mm by 6 atm of pressure. Reproduced with permission from [55,56].

Stainless steel that is used in the biomedical applications is typically classified as the conventional stainless steels or Ni-free stainless steel. Ni-free stainless steels, as the name implies does not possess any Ni content, as nickel, while increasing corrosion resistance also reduces stress corrosion and biocompatibility [53]. Figure 2 shows an example of common allergies associated with implantation of Ni-containing steel implants. Therefore, nitrogen is commonly alloyed with Ni-free stainless steel in order to maintain low Ni content. The nonmagnetic nature of nitrogen does not affect the magnetic resonance imaging (MRI) that is frequently used in biomedical industry. Where conventional stainless steels are primarily used for load bearing applications, both types are routinely used for stents. Figure 3 shows an example of a stent used to maintain the flow of blood in a diseased artery. Even though stainless steel is less corrosion-resistant and biocompatible than titanium, the unique oxygen-rich environment in which the stent is located minimizes the risk of corrosion risk, thus enabling the use of much more affordable stainless steel for this particular application [54]. 

Compared to Ti, biocompatibility, osseointegration and corrosion resistance of stainless steel is considered inferior, yet the cost of titanium is relatively high when compared to stainless steel. For example, 316L stainless steel, where denotation L means that the carbon content in the alloy is less than 0.03%, is merely one fifth of other metallic biomaterials [57], yet exhibits sufficient mechanical properties and ductility. Hence, for bone fracture treatments where complete tissue regeneration is expected, stainless steel is still widely used in the form of screws, nails, and fracture plates to provide temporary support and subsequently be removed surgically after the facture is healed. 

316L Stainless steel can be used to fabricate durable implant trials, i.e., disposable low-cost copies of actual implants which can be used by surgeons during elective joint replacements to determine the correct dimensions of the implant. Traditionally, those were performed with the actual implants, however that approach required repeated sterilisation and decontamination, which was found to not only be inefficient in removing the biological and chemical residue, but also to significantly undermine the mechanical strength of the implant due to sterilisation-induced corrosion and fatigue [58]. While this practice in no longer used in developed countries, the need for correctly identifying the size and dimension of the implant or fixture remains. As discussed, 3D visualisation of the patient and printing of the custom implant is a potentially-effective way to produce correctly-sized implants. A slightly different approach was proposed by Frame [58], who used laser sintering to produce high-fidelity copies of as-manufactured, pre-packaged implants using low-cost 316L stainless steel. Thus fabricated models were amenable to repeated sterilisation with gamma irradiation, ethylene oxide gas treatment and autoclaving, and reprocessing. 

Stainless steel has been used to fabricate 3D dental implants using SLS/SLM, specifically liquid phase sintering, where a binding polymer is melted by a laser beam (at ~1 J mm^−3^) and then used to bind the metal particles. Once printing is completed, the resulting scaffold is heat treated to remove the residual polymer, followed by further sintering and infiltration of bronze to produce an implant of sufficient density [59].

### 3.2. Titanium and Ti-Based Alloys

The performance of medical grade titanium alloys is superior to that of stainless steel due to a 50% greater strength to weight ratio of the former, which makes it a better suited alternative for applications with high loading rates [18]. The weight of the alloy is an important factor that determines that degree of distress to which the adjacent bone will be subjected [60]. The high dielectric constant of titanium dioxide layer that rapidly forms on the surface of bare Ti promotes cell integration, enabling much stronger contact between Ti-based implants and tissues compared to steel. Annealing, quenching and thermal ageing of Ti alloy may be used to further increase the strength of this material [61]. 

Alloying is also commonly used to further enhance mechanical strength of Ti. Among them, Ti-6Al-4V alloy containing 5.5–6.5 wt % and 3.5–4.5 wt % of aluminium and vanadium, respectively [62], is commonly used for it offers better strength profile compared to commercial unalloyed titanium. Addition of Al increases the hardness of Ti by 32% without significantly affecting its other properties [63]. Addition of niobium to Ti, as in Ti_15_Nb_4_Ta_4_Zr alloy, is known to increase both strength and wear resistance, the latter attributed to the presence of a hard, low-friction Nb_2_O_5_ layer [64,65]. However, alloying may also reduce ductility of Ti, which may be undesirable for applications where bending may be a part of the implant function, and for which unalloyed Ti may present a more suitable alternative [63]. 

3D printed titanium implants hold great promise in restoration of anatomically complex areas with functional demands, such as craniomaxillofacial surgery. One-piece 3D-printed titanium mesh implants have been shown to be excellent candidates for a single-operation repair of bifrontal skull defects [66] and maxillary and orbital floor reconstruction [67]. Faithfully rendered, these implants required lower operation time and produced superior aesthetic and functional outcomes, with implants showing suitable long-term stability and absence of trigeminal or facial dysfunction in patients. 3D configuration may also provide improved stability after fixation of fracture, e.g., mandibular fracture, compared to the conventional system of plates and screws due to configuration rather than higher plate thickness or screw length [68]. This may facilitate better blood supply to the bone tissue. Yet, as was shown in the example of the 3D-printed titanium mesh implants, these were still susceptible to subclinical infections that necessitated the administration of antibiotics. The susceptibility to infection was also shown in the 3D titanium implants used in the reconstruction of traumatic zygomatico-orbital defects, which required implant removal [69].

3D printing was also used to produce customized titanium prosthesis in limb salvage surgery to replace bones lost to clavicle Ewing’s sarcoma (ES), scapular ES, and pelvic chondrosarcoma [70]. An electron beam melting system was used to manufacture implants from Ti-6Al-4V powder. This technique overcomes challenges that arise from the excessive chemical affinity of liquid Ti to atmospheric gases and associated significantly reduced ductility of the metal [71]. These implants provided a good match to anatomic structure of the patients, with favorable patient outcomes. Porosity was introduced into the structure of the implant to bring the excessively-high moduli of commercial pure Ti (112 GPa) and Ti-6Al-4V (115 GPa) alloy closer to that of the cortical bone (7–30 GPa) to minimize stress shielding, rather than to promote tissue regeneration or vascularization. Furthermore, the modulus of the resulting structure was not tested in this study [70]. However, previous studies have shown that porous Ti materials fabricated by selective electron beam melting (SEBM) and implanted into the frontal skull of 15 domestic pigs showed abundant bone formation within the implant (at 46% after 60 days) [71].

In addition to SEBM, direct metal laser sintering (DMLS) have been employed to produce titanium-based implants, such as those used to support bar-retained maxillary overdentures. After 3 years, the implant-based and patient-based implant survival rates were at 97.4% and 92.9%, respectively, with the patient-based incidence of biological and prosthetic complication at 7.1% and 17.8%, respectively [72]. Similar cumulative survival rates were reported for DMLS-produced immediately loaded, unsplinted Ti mini-implants providing support for ball attachment-retained mandibular overdentures [73] and 1-piece narrow-diameter SLS implants fixed into the posterior jaws [74]. 

The main advantage of selective last sintering technique is that it is possible to integrate gradient porosity into 3D Ti-6Al-4V dental implants, resulting in a structure rich in interconnected grooves 14.6–152.5 μm in width and 21.4–102.4 μm depth, and a Young’s modulus gradient that varies from ~104 GPa at the metal inner core to ~77 GPa for the highly porous outer shell [75]. Processed in this manner, the inner core is comprised of columnar beta matrix with alpha and beta laths. Ti scaffolds with anisotropic properties, such as porosity and compression strengths (e.g., ~105 MPa and ~25 MPa in the axial and transverse directions, respectively), can also be produced using sacrificial wax templates [76]. However, it must be noted that minimizing discrepancies in architecture between the intended and the produced scaffolds remains a challenge, which may potentially affect both the durability of the implant and the nature of cell-implant interactions [77].

Thus-fabricated dental implants are more fatigue resistant that porous titanium structures obtained using conventional spraying and coating techniques, where fatigue resistance can be reduced by up to 30% by the treatment. Furthermore, SLS enables simultaneous control over the nano- and microstructure within the bulk and the overall geometry of the implant, unlike such techniques as solid-state foaming by creep or superelastic expansion of argon-filled pores, powder plasma spraying over a dense inner core, cosintering precursor particles, or titanium fibers sintering [78]. It should be noted that many of the aforementioned techniques also produce implants with comparable biocompatible and osseoconductive properties [79]. For instance, a study on Beagle dogs implanted with Ti-6Al-4V implants for 1, 3, and 6 weeks showed that significantly improved osseoconductivity and biomechanical response compared to alumina-blasted/acid-etched implants was only observed at certain stages of implantation [80]. 

### 3.3. Cobalt-Based Biometals

Cobalt (Co) based implants have higher wear resistance compared to Ti alloys, which warrants their extensive use in artificial hip joints, where the direct contact between femoral head and the bone or plate over time may lead to wear. Clinically, Co-Cr-Mo is one of the most commonly used alloy due to a favourable combination of high strength and high ductility [55]. When compared to cast Co-Cr alloys, wrought Co-Cr alloys that contain Ni, e.g., Co-Ni-Cr-Mo, have higher strength, however since Ni is potentially toxic, it is only used in those applications where this additional strength is required. The elastic modulus of Co-Cr alloys is also higher than that of commercial pure Ti or Ti alloys [81]. The yield strength and tensile strength of Co-Cr alloys are in the range of 448–1606 MPa and 655–1896 MPa, respectively, whereas Ti-6Al-4V has the yield strength of 896–1034 MPa and the tensile strength of 965–1103 MPa [81,82]. 

Compared to that of bone, the Co-Cr alloys have higher elastic modulus and greater density and stiffness [83], which leads to greater stress shielding than in the case of Ti and Ti alloys or Mg [84]. The biocompatibility of and osseointegration capacity of Co-Cr is also lower than that of Ti. Thus, in clinical settings, it is common for Ti to be used for elements that will be in direct contact with the bone, e.g., screws, and Co-Cr to be the choice material for those elements that do not interface with the bone, e.g., rods in spinal fixation. Yet, such constructs give rise to metal corrosion, in particular considerable mechanically-assisted crevice corrosion, and shredding at the site of the contact between Co-Cr and Ti, which is under significant frictional load. Indeed, the tissue in the proximity of the interface between these materials in total hip arthroplasty, knee implants and spinal fixation most commonly experiences metallosis. Due to superior wear resistance of Co-Cr alloy, Ti is a major source of metallic debris in such constructs. 

Similar to Ti implants, a major challenge that 3D printing can help overcome is the excessive structural stiffness of Co-Cr alloy. By incorporating nano- and micro-geometry within the bulk of the alloy, it may be possible to reduce the elastic modulus and lessen the stiffness discrepancy between the alloy and the bone. Electron beam melting (EBM) is a suitable technique for 3D printing of Co-Cr alloys, which has been successfully used to create Co-Cr implants with desired macro-geometry and bulk interconnected pore architecture [85]. When implanted into adult sheep femora for 26 weeks, these implants showed good total bone-implant contact of ~27%, which was somewhat lower than that observed for Ti-6Al-4V of the same inner and outer geometry. Nevertheless, gradual tissue ingrowth and densification around the implant, as well as mineral crystallinity, apatite-to-collagen ratio, and carbonate-to-phosphate ratio in the formed bone were similar between the two types of implants. The osteocyte density was also higher at the periphery of the Co-Cr porous structure, suggesting a different rate of bone remodelling and a distinct biomechanical environment [85]. 

In addition to foam monoliths, solid and mesh implants can be printed using EBM. Structurally, Co-29Cr-6Mo alloy mesh-strut and foam-ligament microstructures both formed columnar directional Cr_23_C_6_ precipitate architectures spaced ~2 μm in the build direction, reflecting melt pool directional solidification of solid cylindrical components in the assembly process [86]. Solid Ti-6Al-4V implants prepared by the same method displayed α- phase acicular platelets, whereas mesh and foam Ti-6Al-4V implants displayed mainly *α*′-martensite phase with some residual *α* phase. 

When prepared by selective laser melting (SLM), strong temperature gradients during melting and subsequent rapid cooling of the alloy resulted in the creation of a fine cellular microstructure in CoCrMo implant, with grain boundaries depleted in Co and enriched in Mo. The processing also minimizes carbide precipitation and formation of a martensitic *ε* phase at the surface. This provides enhanced corrosion resistance to the 3D printed implant compared to conventional cast alloy [87]. These features also limit the release of the metal ions into the peri-implant milieu, decreasing the risk of metallosis. Both corrosion and ion release rates were linked to the number of laser melt pool boundaries.

### 3.4. Tantalum-Based Bio Implants

**Figure 4:**
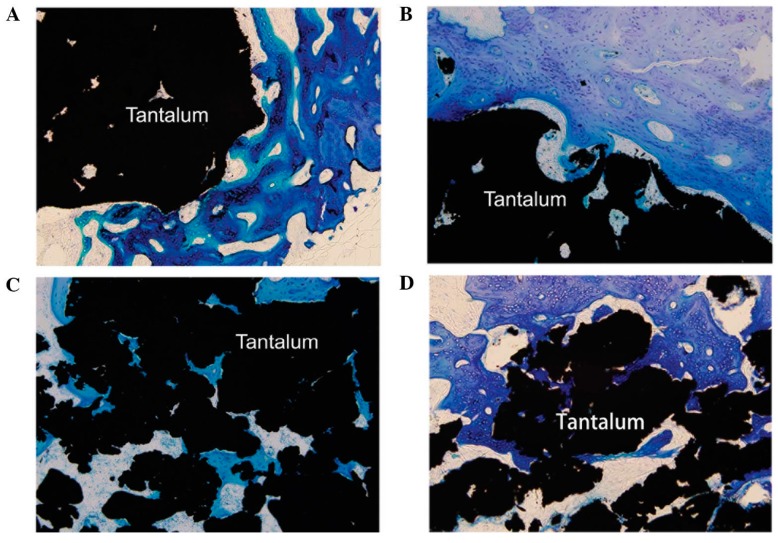
Histological observation of hard tissue in contact with porous tantalum implants at week (**A**) 2; (**B**) 4; (**C**) 8 and (**D**) 12 after implantation (methylene blue staining). Reproduced with permission from [95].

As a biocompatible metal, tantalum has been studied for many biomaterial applications, where biocompatibility and outstanding corrosion resistance even in acidic media are required [88]. The anticorrosion properties of tantalum are due to the stable, native Ta_2_O_5_ protective film formed on the implant surface [89,90]. Porous tantalum has excellent bone-bonding properties, which makes it an attractive material for artificial joints as bulk material or as a coating on stainless steel and titanium implants to enhance corrosion resistance and osseointegration (Figure 4) [91,92,93,94].

However, the wide spread use of this metal is limited by its high elastic modulus and difficulty with manufacturing this material with high precision. The elastic modulus and density of tantalum are above 186 GPa and 16.6 g/cm^3^, respectively. When used in orthopaedic implants, these properties are detrimental, mainly due to the significant difference when compared to those of natural cortical (12–18 GPa) and cancellous bone (0.1–0.5 GPa) [90,96,97]. From manufacturing standpoint, the refractory nature of this metal, specifically very high melting temperature of approximately 3017 °C, makes bulk production of this metal a significant challenge [90]. High temperature conductivity of Ta may also lead the patients to experience temperature-dependent headaches when Ta was used for cranioplasty [98].

Titanium-tantalum alloys containing 50 wt % of Ti and Ta were manufactured into implants using selective laser melting (SLM). Structurally, the alloys were composed of Ti-Ta matrix with randomly-dispersed pure Ta nanoparticles. The matrix was composed of equiaxed grains of β phase Ti and Ta in random orientations, owing to β stabilizing effect and rapid solidification [99]. Compared to commercial pure Ti and Ti-6Al-4V alloy, thus-fabricated Ti-Ta alloy demonstrated higher strength to modulus ratio, with ultimate tensile strength of ~925 MPa and elastic modulus of 75 GPa.

### 3.5. Challenges with Permanent Metals

By now, we have already demonstrated that 3D printing can provide adequate load bearing function for the fractured musculoskeletal tissue [100], showing better match to the anatomic peculiarities of individual patients. We have also discussed the controlled introduction of porosity as the means to match implant Young’s modulus and stiffness to the adjacent cancellous and cortical bone (Table 1), and thus limit stress shielding, a serious issue that often results in re-fracturing of the already weakened bone [100]. Indeed, bone growth and bone density are directly related to loads exerted on that area of bone tissue. Since titanium alloys could be more than ten times as strong as cortical bone [100], its use may lead to drastic reduction in the forces experienced by the bone, which in turn may lead to loss of density and bone weakening [100]. These benefits can be attained while still preserving sufficient compressive strengths and high fatigue resistance, and with the additional benefit of improved osseointegration.

On the other hand, unlike biodegradable ceramics and polymers, inert metallic scaffolds cannot be readily impregnated in situ with bioactive molecules or cells, which somewhat limits the use of these kinds of scaffolds for complete tissue regeneration. Post-processing, such as functionalization of the surfaces with pharmaceutically relevant biomolecules, such as paracetamol [101], attached onto phosphonic acid-based self-assembled monolayers is therefore required to control surface chemistry.

Furthermore, by significantly increasing the surface to volume ratio through addition of interconnected pores, there is a chance that the leaching of metal ions from stainless steel, Ti, and Co-Cr and Ti alloys into peri-implant milieu will also increase. This may be particularly significant when used in environments poor in dissolved oxygen, since surface oxide films are known to inhibit ion release in vivo. Inorganic ions, proteins, and cells present in the body fluids may further accelerate ion release. Stress-induced wear and fretting may promote the removal of the oxide and release of metal ions. Inorganic salts, oxides, hydroxides, and other compounds that form as a result of the reactions between highly chemically-reactive ions and water molecules and anions may also affect cell-surface interactions. The interactions between ions and host cells may lead to the development of sarcoma.

## 4. Biodegradable Biometals

Using biodegradable metals in lieu of permanent metallic implants may produce far better means of fracture fixation for applications where complete tissue regeneration is expected. Currently, magnesium (Mg), iron (Fe) and zinc (Zn) alloys are best researched biodegradable metals for orthopaedic and cardiovascular uses [104], for they offer good in vivo biocompatibility, controlled degradation profile and sufficient mechanical strength to support bone during regeneration process. Indeed, bioresorbable metals have superior mechanical behaviour when compared to bioresorbable polymers, e.g., polylactide (PLA), polyglycolide (PGA) or polylactic-glycolic acid (PLGA) copolymer, since these polymers are brittle and may not be suitable for applications where significant forces are applied to the implant [100,104]. Furthermore, unlike Mg, Fe and Zi, the products of biodegradation of which are naturally metabolised by the host cells, the by-products of polymer break down may lead to inflammatory tissue response and necrosis [102].

### 4.1. Magnesium Alloys

Among biodegradable materials, Mg shows very high specific strength [105], relatively low elastic modulus of 41 GPa and low density of 1.74 g/cm^3^ [106], which are closer in value to those of bone, thus minimising the risk of stress shielding [105]. It can be formed into screws, rods and metal plates, which, once implanted, would provide mechanical support and gradually degrade, providing the space for the growing bone tissue [107]. Gradual degradation would also lead to gradual increase of the force onto the bone tissue, positively contributing to the density and strength of the bone tissue. Eventually, the biodegradable implant would be fully degraded and eliminated from the body, minimising the incidence of metal-related sensitivity observed in the case of permanent Ti and Co implants [107]. 

Although magnesium has promising mechanical and biological properties, it has a rapid corrosion rate in physiological environments, especially in biological fluids [108]. This may lead to large amounts of Mg^2+^ ions being released, and premature loss of mechanical strength of the implanted material [109,110,111]. Having very low corrosion resistance also leads to the release of hydrogen gas which may form pockets of gas around the implant [112]. It is also worth noting that having excessively high corrosion resistance would also hinder bone regeneration. 

**Figure 5:**
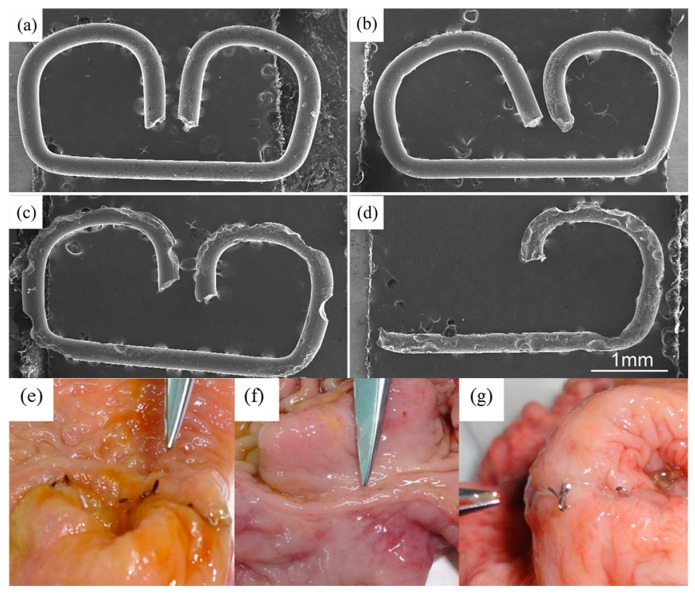
Degradation of magnesium staples under in vitro and in vivo conditions. Optical visualisation of morphology of Mg staples after immersion in simulated body fluid at pH of 4 for (**a**) 3; (**b**) 7; (**c**) 11; and (**d**) 14 days. Photographs of Mg staples implanted into an animal model for (**e**) 7 and (**f**) 90 days; and (**g**) Ti staples implanted for 90 days. Reproduced with permission from [116].

Evolution of hydrogen gas is not a significant problem when Mg is used in stenting since the access of hydrogen is removed by the blood flow. In these applications, the loss of radial strength prior to vessel regeneration is a far more significant issue. Alloying can be used to control degradation kinetics of Mg. A side-by-side comparison of Mg alloy and stainless steel 316L stent in porcine coronary arteries 30 days after angioplasty showed that the magnesium stent induced little inflammatory response, showing an obvious reduction in stent wire thickness and no evidence of thrombosis. Figure 5 shows that Mg staples can be used to replace Ti staples for gastrointestinal anastomosis, since they can maintain their structural integrity for more than 7 days in vitro and in vivo, degrading completely in an animal model. 

Mg scaffolds containing topologically-ordered pore architectures were prepared with indirect solid free-form fabrication (SFF), featuring high resolution and tenability in terms of porosity, stiffness, and volume fraction [113]. The approach included SFF mould printing, NaCl infiltration, and liquid Mg casting [114], and was fairly accurate, with a maximum reduction in porosity and difference in pore dimension of ~6% and ~8%, respectively, and surface roughness of ~10.17 µm [113,115]. The process resulted in a significantly higher surface area available for cell-surface interactions. 

### 4.2. Zinc Alloys

Zinc is another trace elements that plays a significant role in the structure and function of proteins, being essential to catalytic functions in more than 300 enzymes, inducing folding and stabilizing of protein subdomains, e.g., DNA-binding domains of eukaryotic transcription factors, RNA polymerases and accessory proteins engaged in nucleic acid replication [117]. Currently, zinc alloys are being explored for bioresorbable metallic stent applications, since most tissues have good tolerance to excess Zn ions [102]. Anodic dissolution and cathodic reduction of dissolved oxygen are the main processes involved in the corrosion of Zn [102], with pH of the surrounding environment playing an important role. For instance, at pH levels of 7.3, zinc chloride (ZnCl) and zinc oxide (ZnO) begin to participate in the corrosion process [102]. The corrosion rate of pure Zn is lower than that of pure Mg, and is not associated with the evolution of hydrogen gas.

When used as stents in rat arteries for up to 6 months, pure Zn wire did not lead to inflammatory response or extensive thrombosis [102], with evidence of considerable tissue integration within the partially degraded stent. In addition to the use of Zn in stent applications, Zn and zinc alloys (specifically Zn-Mg) are currently investigated for fracture fixation. Combing zinc with magnesium increases the corrosion rate whilst also improving the mechanical properties of the alloy [104]. Yet, further investigations into the corrosion behaviour of these alloys in vivo are warranted to fully understand the corrosion mechanisms, such as the role of the surface oxide layers in biodegradation and cell-surface interactions [29,104]. 

### 4.3. Iron Alloys

Similarly to Zn implants, Fe alloys undergo local corrosion mediated by dissolved oxygen, a process that does not produce any hydrogen gas [102]. Among biodegradable metals, iron has the lowest tendency to dissolve, with the degradation rates comparable to that of arterial remodelling [104]. Low rate of corrosion is in part attributed to the formation of a protective surface oxide layer which acts as a barrier to prevent rapid degradation [104]. Several studies using Fe bio-resorbable stents in rabbits, pigs and rats have shown no significant negative inflammatory response, excessive toxicity or thrombosis [102]. The Fe stents exhibit high radial strength and therefore allow for extremely thin stent struts, producing a more ductile structure and making it easier to deploy into the artery [102].

Although the superior mechanical properties and slow corrosion process are positive attributes, they may also produce some undesirable consequences. For instance, incomplete corrosion within the follow-up period has been reported for Fe stents, suggesting the need to for increasing the degradation rate though alloying [102]. Yet, production of iron oxide degradation by-products and influx of metallic ions into the surrounding tissue also need to be carefully controlled. The recommended daily intake of iron is 6–20 mg but the long-term effects of iron overdose can include inflammation, increases in free radicals and damage of lipid membranes, proteins, and DNA [4,102]. 

Due to its magnetic nature, presence of iron in surgical implants may potentially interfere with Magnetic Resonance Imaging (MRI), which is commonly used to visualise the anatomy and the physiological processes of the patient during diagnosis, healing, and follow-up observation. Exposure to strong magnetic fields may also lead to heating of the implant, potentially causing it to change its shape or position. It should be noted that these effects can be harnessed to enhance therapeutic outcomes of the treatment. For instance, porous magnetic scaffolds containing iron nanoparticles have been shown to stimulate osseous tissue generation by attracting growth factors, hormones and polypeptides, and promoting cell adhesion and proliferation. Upon application of an external magnetic field, Fe particles are displaced, applying compression and tensile forces on the attached cells and thus inducing cytoskeleton deformation and cell dragging, and activating intracellular signaling pathways associated with natural processes of bone formation [118]. Application of external magnetic field can also be used to trigger drug release, whereas localized heating may be used for thermal therapy of cancer and implant-associated infections. 

For orthopaedic application, excessively slow degradation rate may hinder tissue regeneration [4], and limit the transfer of forces to the growing bone, resulting in stress shielding, which in turn results in reduced bone density and cross sectional area. Similar to permanent implants, the mechanical properties of iron offer an inadequate match to those of a natural bone. Stress shielding can be reduced by introducing controlled porosity into the bulk of Fe implant [4]. The increased surface to volume ratio may also increase the degradation rate of this metal, bringing it in line with respective rates of regeneration for bone and vascular tissue. Furthermore, the porous structure fabricated by 3D printing may be designed to enhance osteogenesis, and can be loaded with grafting materials, engineered tissues, or drugs. 

Introduction of micro-pores within implants fabricated suing binder-jet 3D printing from Fe-Mn-1Ca and Fe-Mn alloys have been shown to increase the rate of corrosion in both alloys [4]. With a typical diameter of approximately 5 µm, the micro-pores also enhanced cytocompatibility. Compared to Fe-Mn, microporous Fe-Mn-1Ca alloy exhibited higher stiffness and ultimate tensile strength [4]. Introduction of micro-pores resulted in the embrittlement of Fe-Mn-1Ca alloy, which may limit their use in some load-bearing applications.

## 5. Limitations of Biomaterials and Strategies for Enhancement

With the increased need for synthetic limbs, joints, and other body part replacements, it is important to be aware about the limitations of these materials that could be utilised for these prosthetic body parts and existing strategies to overcome these limitations. 

### 5.1. Biocompatibility

Although currently-used permanent and biodegradable metals are general biocompatible, excessive wear and premature degradation may negatively affect their biocompatibility, hinder healing and cause long-term damage [119]. Furthermore, methods commonly used to enhance mechanical or corrosion resistance properties of the material may reduce biocompatibility, e.g., addition of Al or Ni is known to increase the incidence of inflammation in the surrounding tissues [120]. To some degree, this issue can be mitigated by minimising the direct contact between cells and the implant, such as in the case of Co-Cr rods which can be affixed to the bone using more biocompatible Ti screws [121]. Strategies for prevention of bacterial contamination can also alter host cell-surface interactions, potentially hindering osseointegration [122,123]. 

**Figure 6:**
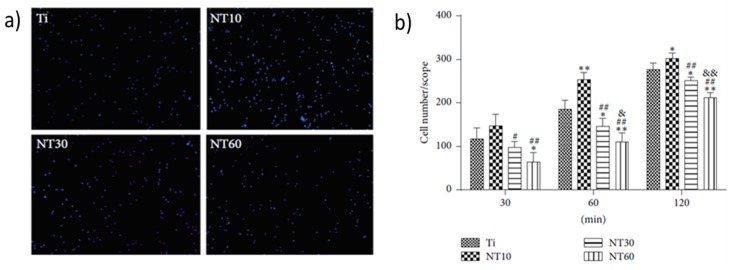
Fluorescence images of initial adherent bone marrow mesenchymal stem cells (BMSCs) stained with DAPI after 1 h (**a**) and cell numbers measured by counting cells for 0.5 h, 1 h, and 2 h (**b**). Cell attachment on NT10 (titanium nanotube with 10 nm diameter) was significantly improved compared with control Ti surfaces for 1 h and 2 h. In contrast, cell attachment was inhibited on NT30 (titanium nanotube with 30 nm diameter) and NT60 (titanium nanotube with 60 nm diameter) at each time interval adopted in this study. All data were expressed as the mean ± standard deviation (SD) and ‘*p*’, the level of significance. *p* < 0.05 was considered significant, and *p* < 0.01 was considered highly significant. Here, the mean ± SD N = 3, and * *p* < 0.05, and ** *p* < 0.01 compared with the T; ^#^ *p* < 0.05 and ^##^ *p* < 0.01 compared with the NT10; ^&^ *p* < 0.05 and ^&&^ *p *< 0.01 compared with NT30. Reproduced with permission from [124].

Surface modification of these implants is one of the most common strategies to enhance surface biocompatibility. Figure 6 depicts the reaction of bone marrow mesenchymal stem cells (BMSCs) on differently-modified titanium surfaces [124]. Here, titanium nanotubes were created on a titanium sheet, cleaned in nitric acid and left to dry. The Ti was then used as an electrode and a sheet of platinum was used as a cathode during anodization in 0.50 wt % NH_4_F + 10 vol% H_2_O in glycerol at 10 V, 30 V and 60 V for 5 h, followed by annealing. Among these surfaces, Ti nanotubes that were subjected to a lower voltage during anodization showed highest biocompatibility, characterised by higher growth and stronger attachment of bone mesenchymal stem cells (BMSCs).

**Figure 7:**
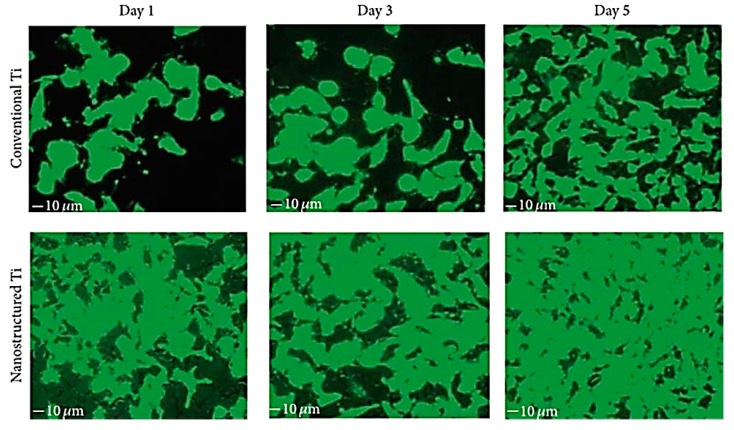
Fluorescent microscopy images of endothelial cell proliferation on nanostructured Ti compared to conventional Ti. Reproduced with permission from [129].

Addition of surface roughness and porosity stimulates cell attachment and osseointegration. Traditional methods range from sandblasting to acid etching to laser ablation. For instance, sandblasting of Ti with large grit acid can be used to create roughness over the implant surface, providing micro-level topography for growth and attachment of cells [125]. Nano-scale topography has been shown to improve wettability, which increases adhesion of cells. Figure 7 shows the effect of nanostructured Ti, where greater adhesion is evident on the surfaces of nanostructures rather than conventional Ti surfaces [125,126]. However, it must be noted that these changes to surface topography may also have a direct effect on the nature of interaction between pathogenic microorganisms and surfaces [127,128]. 

In addition to surface modification, bulk modification can be used to enhance biocompatibility, such as alloying elements that enhance cell attachment and proliferation, changing grain structure, or introducing of controlled porosity. As already discussed, in addition to conventional methods such as gas foaming, solvent casting with particle leaching, freeze-drying and electrospinning, and in particular, 3D printing is one of the most promising methods for introducing complex interconnected architectures and curved channels with controlled distribution into metallic materials [39,40]. However, it is important to note that by significantly increasing the surface area which is in contact with potentially-corrosive implant milieu, it is possible to change the degradation profile of the material. This may substantially increase the amount of ions which are released from the implant, which, in some instances, can lead to the development of sarcoma [46]. It may therefore be necessary to coat the surface of the 3D scaffold with a thin film of polymer or ceramic, that can serve to both limit ion leaching and enhance cell attachment and infiltration. 

It is important to select a surface modification technique that would not compromise the desired dimensions and connectivity of internal pores and channels. For conventional functionalization, solvent casting and dip-, spray- or spin-coating are frequently used, and may in principle be compatible with metal scaffolds. However, thin films typically produced by these techniques suffer from non-uniformity with regard to the type and distribution of chemical functionalities, hydrophilic or hydrophobic domains, and surface roughness [101]. Such non-uniformity in thickness can negatively affect the finely-resolved internal structure of the implant. Furthermore, polymer thin films may be subjected to fissures, cracks and waviness, which may result in local inflammation. 

To maintain the desired level of precision, an approach that provides sufficient degree of control over the distribution, orientation and attachment of the functional groups or active molecules is critical. Self-assembly offers better control over the spatial distribution, producing a monolayer that is highly suited for functionalization with active molecules, e.g., growth factors, hormones, polypeptides, drugs, antibodies etc. Importantly, the monolayer is typically several nm thick, preserving the internal geometry of the 3D printed structure. Furthermore, the chemistry of the monolayer can be altered for specific application. The assembly itself is a relatively simple process, which does not interfere with the bulk materials properties of the underlying material. 

### 5.2. Surface Colonisation and Biofilm Formation 

Implants that selectively prevent attachment and growth of bacteria and inhibit biofilm formation without comprising the favourable host cell-surface interactions are highly desired. When an artificial implant is introduced into a body, protein attachment ensues, followed by a competitive process where host and pathogenic cells attempt to colonise the surface [130]. In many instances, strategies that delay microbial colonisation for even a short period of time are sufficient to provide host cells with competitive advantage. However, in other instances, where host cells do not colonise the surface, longer-term antimicrobial strategies are necessary to prevent biofilm formation [131,132]. One of the widely used methods is to coat the surface of the biomaterial with antibacterial agents at doses that are safe to host cells. Silver nanoparticles are widely used antibacterial coating, with silver nanoparticles-coated Ti-based biomaterials showing excellent antibacterial properties [132].

Plasma-enabled biomedical technologies have emerged as a promising approach for non-chemical, low-temperature decontamination in the biomedical, food manufacturing and food service industries, and synergistic and personalised plasma-enabled therapeutics for tissue regeneration and oncotherapy [133,134,135,136]. There is a rapidly increasing body of literature where low-temperature plasmas have been used to rapidly disinfect living and abiotic targets, drive cell differentiation and migration, and promote tissue regeneration and wound healing, and, in cancer therapy, to reduce or fully eliminate tumours, halt metastasis, enhance local and systemic immune function and selectively induce apoptosis in cancer cells. Plasma takes advantage of the unique chemical and physical features of the fourth state of matter to expose living or abiotic targets to a controlled mixture of highly chemically-reactive species, electromagnetic radiation and heat to modulate their properties [137]. 

When applied directly to cells or tissues, plasma treatment can alter their cellular activity in prokaryotes and eukaryotes, and thus control the processes fundamental to biofilm formation, tissue regeneration, and carcinogenesis [138]. More intense treatment may be used for surface sterilization and decontamination, whereby plasma etching can remove biomolecules, such as proteins, pyrogens or extracellular polymeric substances, as well as cells and their spores from biomaterial surfaces at high rates and low temperatures. In addition to highly efficient removal of biological matter from implant surfaces, plasma treatment can be an effective tool for lasting, highly controlled surface modification [139], including chemical functionalisation, deposition of antibacterial thin films and coatings [140,141,142,143,144], and surface structuring to create antifouling surfaces. Plasma can also be used for a deposition of highly-complex, ordered surface nanostructures from a wide range of materials [145,146], which can afford a higher level of control over the attachment behaviour of cells and microorganisms, providing a more selective control tool. 

Despite challenging tortuosity of the porous structures, plasma deposition has been successfully used to impart chemical gradients inside 3D porous scaffolds to enhance cell viability when compared to untreated materials [147,148,149]. By varying the processing gas, it is possible to add N- or O-functional groups from NH_3_, N_2_, O_2_, and CO_2_, alkyl amines, acrylic acid, allyl alcohol, ethylene and their combinations, and deposit or etch nano- and micro-scale features on the surface and throughout the entire porous structure [147,150,151].

### 5.3. Wear of Metallic Implants

Despite their relative durability, in the US approximately 1 million total joint implants are expected to fail after 15–25 years of use [152]. Degradation of implants is initiated from mechanical wear of the components, and through electrochemical corrosive reactions with body fluids. Mechanical wearing is most prominent in Metal on Metal (MoM) implants, particularly in hip based prosthetics. A study into MoM wearing defines four wear mechanisms, namely adhesive, abrasive, fatigue and tribochemical wear. Adhesive wear is the process where a segment of an opposing component is ripped away due to local chemical bonds between the surfaces. This results in the formation of pit indentations and scratches along the impacted surface. Additional surface wear is attributed to abrasive wearing whereby asperities are pressed and dragged along the opposing component surface; contact of asperities causes scratches and cuts, but can also polish the surface reducing its surface roughness and thus rate of wear. Abrasion also removes oxide layers and other protective films.

Fatigue wear often takes place when repetitive, cyclic loading on the implant weakens the surface to produce cracks, eventually leading to fragmentation and pitting. Additional wear damage caused by these mechanisms include gouges, etches, surface discolouration, surface deposits and third-body particulate generation [153]. The fourth mechanism is tribochemical, an interplay of mechanical and corrosive wear, caused by mechanical activation of the body’s surrounding fluids [153]. A study into Ti-6Al-4V hip prosthetics showed 90% of surface fractures were caused by both cyclic stresses and corrosion [4]. Tribocorrosion is evident on Ti alloys. Here the corrosion acts as a degradation accelerant due to mechanical removal of protective oxide layers, as seen on cyclic loaded die-cast AZ91D implant which experienced degradation at 7–8 times the normal rate [4].

Corrosion itself is an electrochemical reaction on the metal’s surface, with the bodily fluids acting as the electrolyte. Two reactions occur for corrosion to take place: first is oxidation of the metal surface via removal of electrons and second is reduction of the oxidizing agents which consumes electrons; both processes alter material properties [154]. These reactions are driven through thermodynamic forces of the metal which allow hydration of the metal and subsequent migration of metal ions from the surface, generating metallic debris. Thermodynamic forces determine the potential of a material to corrode, hence Co, Cr, Fe and Ti common use in orthopaedics as they are low on the electrochemical series. Ti and Co specifically are labelled as passive materials as they generate a thin metal oxide barrier of roughly 1–10 nm in thickness [154].

**Figure 8:**
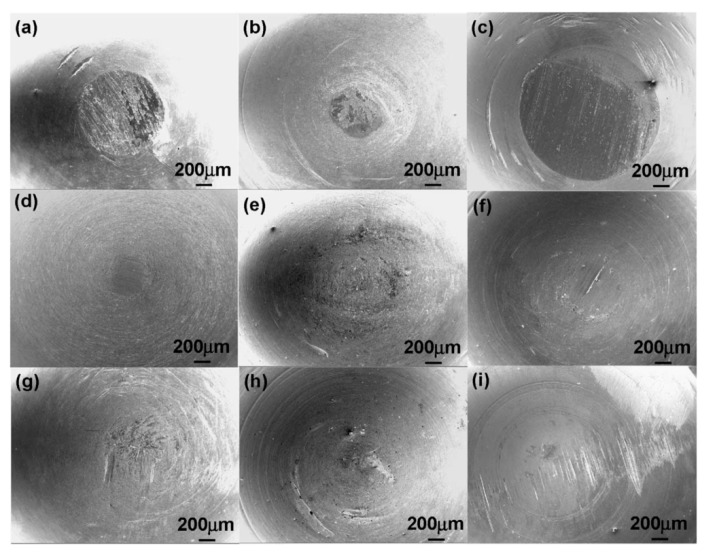
Wear morphology of two differently-processed Ti-29Nb-13Ta-4.6Zr (TNZT) alloys (**a**) TNZT1 and (**b**) TNZT3 and (**c**) a Ti-6Al-4V alloy (TAV1) sliding on a stainless steel plate; (**d**) TNZT1; (**e**) TNZT3; and (**f**) TAV1 sliding on ultra-high-molecular-weight polyethylene (UHMWPE); (**g**) TNZT1; (**h**) TNZT3; and (**i**) TAV1 sliding on a pig bone in 0.9% NaCl. Reproduced with permission from [81].

Degradation of oxide films via chemical or mechanical means allows corrosive fluids to interact with the exposed metals. Figure 8 illustrates changes in surface morphology as a result of mechanical wear. The newly exposed metal has a different corrosion potential to the surrounding body fluids due to higher oxidation kinetics [155]. This creates galvanic couplings between the metal (anode) and the electrolyte fluid. Corrosive wear then degrades the metal through various modes. Pitting, previously labelled as mechanically produced indentations, is a mode of corrosion propagating through localised dissolution of the metal surface. Stress corrosion cracks (SCC) then propagate around the pits from cyclic stresses, resulting in fractures. Alloyed metals within the implant can undergo galvanic corrosion depending on composition and surrounding elements. Biological corrosion mechanisms such as inflammatory cell induced corrosion (ICI) take effect; cells in direct contact with implant attach to the metal surface as a result of inflammatory reactions and degrade the surface. 

Degradation by mechanical and chemical processes weakens the implants structural integrity and thus its ability to function. The four wear mechanisms of the MoM implants, namely scratching, etching, pitting and weakening the structure, increases the surface roughness which in turn increases wear rate [153]. Surface/subsurface corrosion of Mg alloys in particular releases hydrogen gas which induces SCC into the metal. If formed at the tip of crack propagation, hydrogen additionally reduces cohesive strength of the Mg alloy leading to embrittlement of the material and consequent loss of mechanical strength. Mechanochemistry is another form of mechanical wear, where reduction in atomic coherence energy is caused through applied stresses increases metal surface energy [4]. Lowered atomic coherence equates to weakened chemical bonds, making the metal vulnerable to applied stress and corrosion. 

Dissolution of the metal surface through corrosion negatively affects the properties and structure of the implant, reducing its functionality. Oxidation of the materials weakens chemical bonds and can alter properties of the surface and bulk metal. This altering of mechanical properties can also occur through absorption of phosphates, oxygen, chlorides and biological species in the surrounding environment [154]. Migration of metal ions from the surface removes passive layers and material; the former eliminates the implants corrosion protection and the latter weakens the surface structure, making the implant more susceptible to stresses. 

Degradation of metallic implants releases metallic debris, in the form of nanoparticles and solubilised ions; the former being produced from mechanical wear and the latter via corrosive decay [156]. Biomedical metals exhibit varying levels of toxicity within the body and show patterns of accumulation. Mo and Nb debris have been shown to target the brain and lungs after spreading, specifically from hip endoprostheses [157]. Implants containing elements of Ag, Cr, Fe, Mo, Ni and Ta show large concentration of metallic debris in tissues that are in direct contact with the implant. 

Co, Cr, Sb and Sc are also found to accumulate in other organs including the heart, kidney, liver and spleen [157], where they are transported through synovial fluids, the bloodstream and lymph [157]. A 100-fold increase in blood Co and Cr levels were shown in patients who received implants containing these elements. Elevated levels of Ni, Ti, and V were also reported in patients with both failed and successful total joint replacements. Interestingly, Co is distributed evenly into the blood cells and plasma, displaying significant absorption by the body. Results also show elevated metal levels present within urine samples. Increased concentrations of Co, Cr, and Mo were also detected in hair strands of patients with arthroplasties implants, which are known to generate abundant debris [157]. 

Metals of Co, metallic Ni, Ni/Cr alloys and Pb are potentially carcinogenic to the body. Tumour generation is rare, however an experiment on Ni implants in rats saw sarcomas produced at the implant-site. From this it was concluded that the potential of tumour growth is related to the extent of carcinogenic metals allowed to be released into the body [158].

Metal debris can also interact with innate immune cells, i.e., macrophages, producing cytokine and chemokine-based pseudo diseases [152]. The activation of this innate immune system, and subsequent interaction with debris, could then create metal sensitivities, or allergies [152]. This is problematic given prominent use of metals in orthopaedics, vascular and dental implants. Ti particles specifically activate macrophages which release inflammatory cytokines, and initiate a cascade of biological events that eventually lead to osteolysis, forming along the bone/implant interface [158]. 

Wear resistance of the biomaterial has a vital role in the mechanical functioning of the implant, where degradation can result in the loosening of the implants in the body and deposition of wear debris in tissue can lead to inflammation [81]. In addition to various processing techniques and compositional changes, surface modification of the biomaterials can also improve its wear resistance [81]. Ion implantation or physical deposition is one among the few techniques to improve the wear resistance of a biomaterial. Here, the physical or chemical properties of the surface are improved by embedding beam of ionised particles into the surface of the material [81,159]. Other methods which are used to improve the wear resistance of the biomaterials include nitriding, which is used to increase the resistance of an alloy to dry wear [160], carburization and boriding are used to increase the wear resistance by increasing the surface hardness of the implant [81].

Ion implantation is a surface treatment during which a stream of charged particles is accelerated towards a material [161] causing them to collide and altering the surface properties of the material. The beam of ions can affect the material in several ways, i.e., the ions can impact the surface causing changes in its atomic structure owing to formation of new phases and generation of stresses at the interface between the top layer and substrate, which result in generation of dislocations and stress evolution in metals in a similar way to shot peening [162]; through beam mixing where the beam of ions is used to mix an applied film into the base material [162]; and by chemically changing the surface where the ions implanted into the surface to create new compounds that benefit the surface properties of the material. 

The benefits of ion bombardment include reduced cost compared to the development of new materials, preservation of favourable bulk properties of the material. It is also highly repeatable and can be applied to finished parts [163]. The process is flexible by controlling the type of ion used, increasing and decreasing the onset and rate of corrosion [164], changing biocompatibility of the surface [161] and changing the hardness, although in high wear applications the modified surface can easily be worn away [163]. Another advantage of this method is that it can be applied directly to a finished part. The ions used are high energy, between 20 and 200 KeV, but have very low mass so do not penetrate more than a few microns below the surface and thus do not affect the base properties of the material [163]. The processing can be done at temperatures less than 150 °C [162], meaning it will not affect bulk properties, such as the temper of steels, and is done under high vacuum to minimise contamination [163].

**Figure 9:**
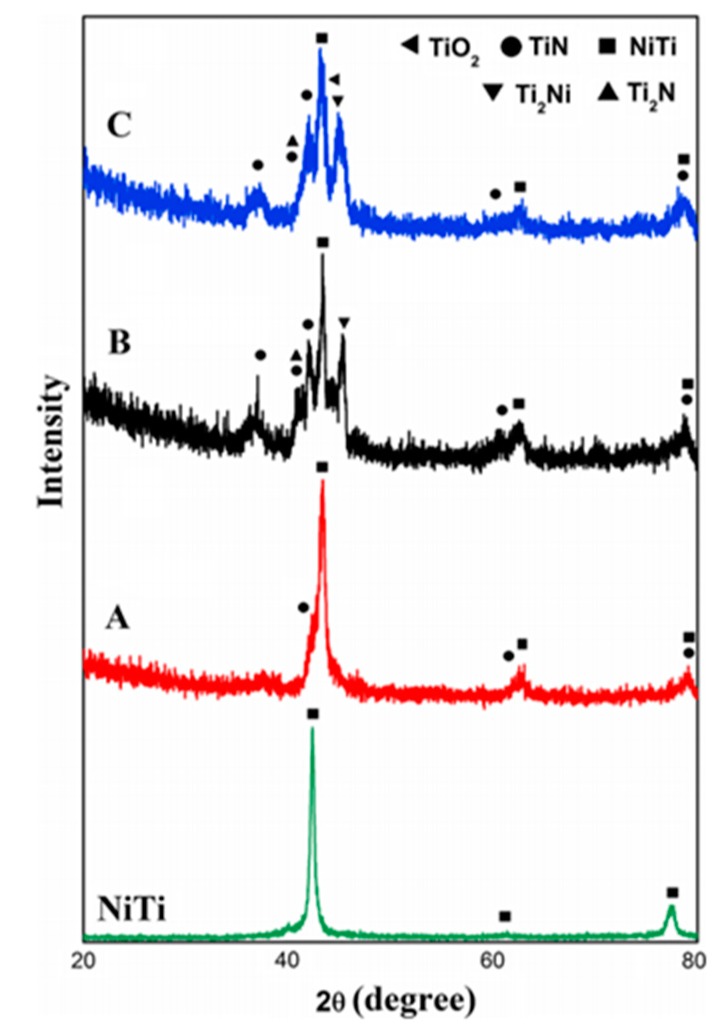
Low angle XRD phase analysis of NiTi surface implanted by nitrogen ions: A, B and C indicated the intensities at different concentration of nitrogen, such as 6.9 × 10^17^ ion cm^−2^ (sample A) 1.4 × 10^18^ ion cm^−2^ (sample B), and 1.8 × 10^18^ ion cm^−2^ (sample C). Reproduced with permission from [161].

Figure 9 shows the effect of N ion implantation on the performance of NiTi alloy. The treatment produced new compounds such as TiN and Ti_2_N that were not present in the original surface, reducing corrosion rates [161] and improving wear resistance [165]. The increases surface corrosion resistance was tested and found to have leached 14 times less Ni ions than the control TiNi sample over an 8 week period [161]. Improved surface properties of the alloy after treatment make it a much more desirable material to use for implants because of the increased bio compatibility and wear resistance due to the decreased leaching of Ni ions. Ion bombardment has also been used to form Ti carbides on the surface, that have increased wear and corrosion resistance compared to TiNi alloy, and limit the leaching of Ni from TiNi implants [161].

**Figure 10:**
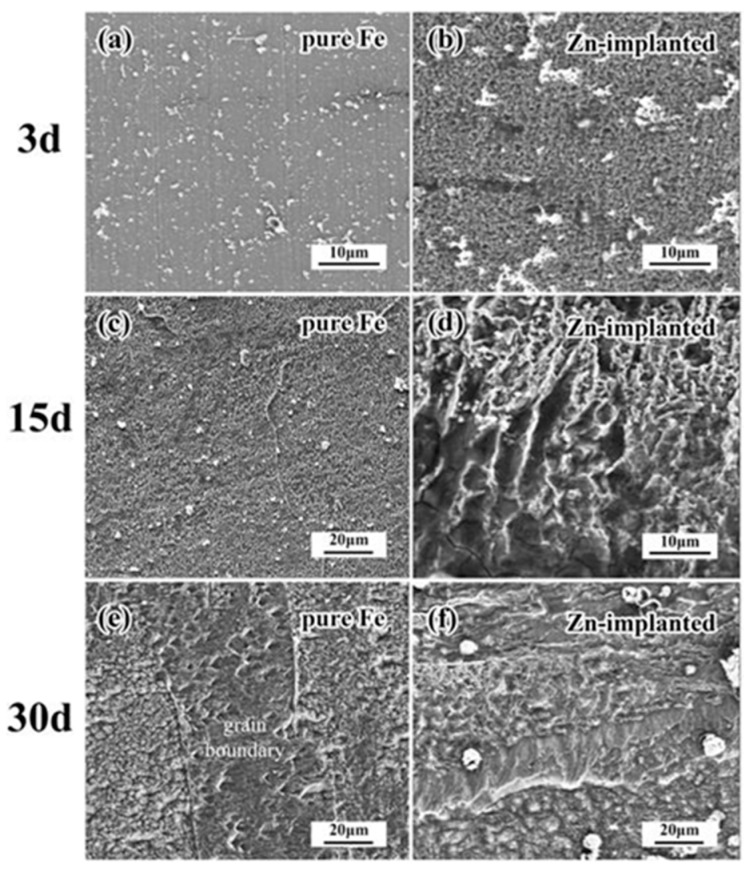
SEM images showing the corrosion after 3, 15 and 30 days. The image shows the increased corrosion rate of the Zn- implanted iron (**b**,**d,f**) in comparison to the pure iron (**a**,**c,e**) samples, with pure Fe samples degrading mostly along the grain boundaries (**e**). Reproduced with permission from [164].

Implantation can be used to increase or decrease the time taken for corrosion to start, which can be extremely useful in controlling biodegradation kinetics of resorbable implants, such as increase degradation rate of iron [164]. When Zn was implanted into the surface of iron, it resulted in the increased rate of corrosion and created more evenly distributed corrosion pattern, as shown in Figure 10. Pure iron, after 30 days, had mainly corroded along grain boundaries. The implantation of zinc ions also changed the biocompatibility of the stents by reducing the attachment of erythrocytes but increasing the adhesion of platelets [164]. The disadvantages of ion bombardment include the fact that ions can only be implanted in line of sight [162], and have extremely low implantation depth and high initial cost of machinery [163]. Since this technique can only change the parts of the surface that are in direct line of sight of the ion beam, it may have limited utility for processing of 3D printed implants and other medical implants with complex external geometries, which require more than one ion exposure. For implants with deep holes or overlapping parts, it may not be possible to achieve the desired level of modification with this technique. Another potential disadvantage is the very small penetration depth of ions, often less than one micron in depth. Thus, even if the surface has been hardened, high wear application could easily wear the surface down.

### 5.4. Mechanical Failure

**Figure 11:**
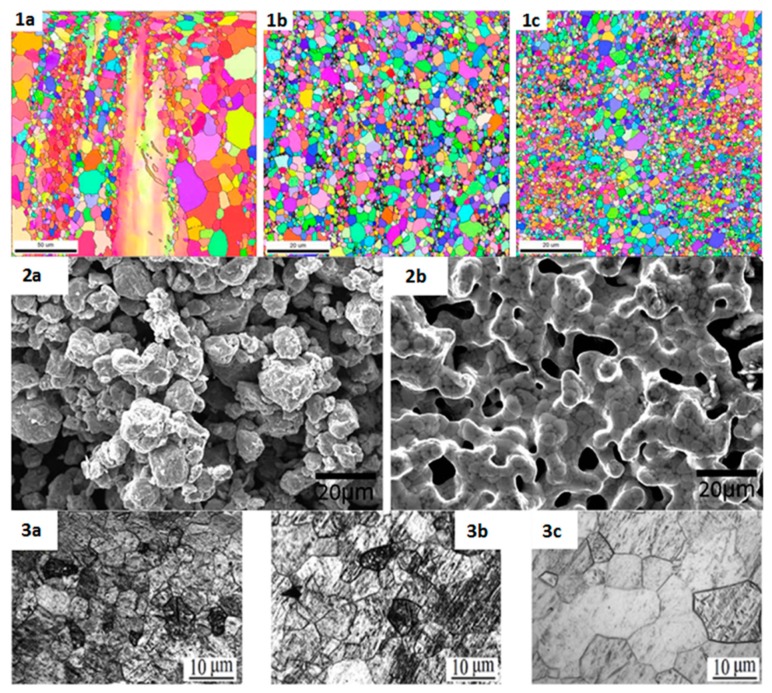
Grain structures or morphologies of magnesium alloy (AZ31) (series 1), iron alloy (Fe-Mn-1Ca) (series 2) and commercially pure (CP) titanium (series 3). Series 1 depicts the cyclic extrusion compression (CEC) process of AZ31 at 300 °C, (**1a**) showing the alloy “as extruded’, (**1b**) showing the alloy after 7 passes and (**1c**) showing the alloy after 15 passes. Series 2 shows the morphology of the iron based alloy before (**2a**) and after (**2b**) the binder-jet-3D printing process has been completed. Series 3 shows the morphology of a CP titanium sample after 3 laser shock peening impacts. (**3a**) shows the morphology of the sample close to the surface, while (**3b**) shows the morphology of the sample less than 0.5 mm below the surface and (**3c**) shows the sample morphology at about 2 mm below the surface. All images—series 1, 2 & 3—were reproduced with permission from [166], [4] and [3], respectively.

Traditionally, physical modification techniques that can be used to significantly enhance properties of biometals include grain refinement, inducing stacking faults, secondary phase strengthening, changing microstructure, and so on. For example, the biodegradation profile of Mg can be substantially altered by the combined processes of hot extrusion, semi-continuous casting with immediate cooling and heat treatment [11], or multi-axial forging (MAF) of as-cast and annealed samples of magnesium based alloys [5]. Figure 11 shows examples of the effects of different processing methods on the grain structure and morphology of commonly used metal alloys.

Magnesium is much easier to synthesise into the complex shapes required for many implants via machining, welding, casting, forming at an elevated temperature, and other conventional methods [84]. As already mentioned, the major issues with Mg implants include fast corrosion, and significantly lower yield and ultimate tensile strength than titanium and iron based alloys. These can be addressed by grain refinement, which can increase mechanical properties without embrittlement, unlike methods such as secondary phase strengthening [11]. Grain refinement procedures also often allow the metal to maintain the elastic modulus, thus allowing Mg to maintain the level of stress shielding low [84]. Grain refinement techniques have been investigated predominantly through manufacturing methods that involve intense plastic deformation. An example of this is a study done by Nayak and colleagues [84], who achieved grain refinement by rolling Mg alloy samples using a bi-directional rolling device, whilst maintaining the sample’s temperature at 310 °C. For every rolling cycle, a decrease in average grain size, increase in hardness, increases in yield strength and ultimate tensile strength, increases in toughness and ductility, relatively constant elastic modulus and an increase in corrosion rate were observed.

In another example, grain refinement of Mg alloys was achieved by cyclic extrusion compression (CEC) [166]. A decrease in grain size correlating with the decrease in extrusion temperature was observed, indicating an optimum temperature and number of cycles at which the CEC should be performed. A high density of dislocations in the samples was also shown suggesting that that the CEC processing of the magnesium alloy would have increased its yield and ultimate tensile strength, hardness, ductility, and toughness. 

Multi-axial forging (MAF) of magnesium alloys at 450 °C manufactured by casting or casting and annealing decreased the grain size with the number of passes [5]. The yield and tensile strength, as well as hardness improved with the number of cycles. Ductility and toughness appeared to be more dependent on the combination of MAF passes and annealing. The sample which showed the smallest average grain size, and overall highest yield and tensile strength, ductility, toughness, and hardness was cast and annealed with 2 passes of MAF. 

These three examples all show how mechanical properties of magnesium alloys can be significantly improved through grain refinement, with results showing yield strength improvements by over 300% and ultimate tensile strength improvements by almost 200%, as well as a 264% increase in toughness [5]. Yet, it is worth noting that multi-axial forging of Mg also increased the rate of corrosion, which is already high in this type of implants. 

Using a combined processes of hot extrusion, semi-continuous casting with immediate cooling and heat treatment, the corrosion rates were reduced in Mg-8Er-1Zn to 0.34 mm/year by inducing stacking faults within the alloy [11]. Both in vivo and in vitro testing showed the mode of corrosion in the Mg alloy to be uniquely uniform because of the stacking faults. The stacking faults also positively contributed to the mechanical properties of the alloy, with yield and ultimate tensile strengths of the investigated alloy being 207 MPa and 318 MPa, respectively, and an elongation of 21%. 

Mechanical properties and corrosion resistance of permanent implants, such as commercial pure Ti can also be improved using bulk modification. Indeed, compared to Co-Cr implants, Ti displays relatively low strength and micro-hardness, and higher susceptibility to wear corrosion [3]. Surface treatment techniques are often used to cause severe plastic deformation at a high strain rate in order to refine grain size near the surface and induce certain microstructural features, e.g., micro-twin grating, micro-twin collision in the compression deformation zone, layered slip band in the tension deformation zone and inverse transformation martensite [3]. This is known typically to enhance mechanical properties such as hardness, significantly increase the fatigue resistance of the metal and improve corrosion resistance. Common surface treatments include laser shock peening (LSP), high energy shot peening, water cavitation peening, cold rolling, and surface mechanical attrition treatment (SMAT) [3]. 

A study on the effects of laser shock peening on CP titanium was conducted by Lu and colleagues [3], who examined and compared the CP titanium micro hardness and microstructure before and after the LSP surface treatment had been applied. The researchers noted that the laser shock peening dramatically reduced the average grain size (from 10–15 µm to 0.05 µm). A ~40% increase in surface micro hardness was also observed after 3 LSP impacts, and a thickened layer around 650 µm deep had formed on the surface. The significant increase in hardness was attributed to the intense grain refinement (nano-crystallisation) and the formation of inverse-transformation martensite. Surface mechanical attrition treatment of titanium using alumina balls was shown to increase surface roughness, hardness, and micro-strain as well as decrease grain size near the surface [167].

## 6. Emerging Metallic Biomaterials and Future Trends

### 6.1. Bulk Metallic Glasses 

A new class of metallic biomaterials are bulk metallic glasses (BMGs) [15]. BMGs, which show no long range atomic order, display some attractive mechanical, chemical and physical properties when comparing it with the traditional alloy. Conventional alloys have a crystalline structure compared to the no long range atomic order shown in BMGs. This makes a BMG alloy have an atomically frozen liquid-like appearance [168]. The glass-forming ability (GFA) of an alloy is the ease at which it can be produced in an amorphous form, by cooling from the liquid. If the GFA is high, it indicates a low required cooling rate. The structure of BMG lacks dislocations and hence slips planes, leading to excellent strength and elasticity. BMGs also exhibit high toughness compared to crystalline metals and significantly reduced corrosion rates due to their isotropic and homogenous nature and lack of grain boundaries or precipitates as oxidation sites [15,168]. 

BMG alloys can be fabricated from bioinert, e.g., Ti, and bioresorbable, e.g., Zn and Fe, metals, and, in comparison with conventional alloys, have superior properties to current biometals such as high strength, low elastic modulus and a high elastic strain limit. The latter properties provide a better match to cortical bone and therefore a reduction in the stress shielding when used in hard-tissue prostheses. Bioinert BMG devices are resistant to corrosion wear and have controllable surface topography. They could be used in orthopaedic prostheses, surgical scalpels and flexible vascular stents [15,168].

Compared to conventional alloys, bioresorbable BMG alloys have more desirable rate of corrosion. The strength decay is inversely proportional to wound healing, i.e., osteogenesis, and thus limits stress shielding. The ions that are released in this process are non-toxic. Once the process is complete, a removal surgery is not needed as the device has been absorbed. Potentially, bioresorbable BMGs can be used as a bone screw or surgical plate, intramedullary nails or a temporary vascular stent. 

Current challenges with bulk metallic glasses include lack of ductility, lack of understanding about crack propagation and mechanical behaviour, e.g., fatigue, the mechanisms of stress corrosion and wear debris behaviour. This limits material optimisation and matching of specific GFA with clinical needs and biocompatibility [15,168].

At present, a variety of shapes that can be produced from amorphous alloys are limited to simple geometries, e.g., foils, plates and rods with thin section-thickness. This is due to the requirement of high cooling rates. 3D printing using recently developed laser foil printing (LFP) may be one of the several technologies capable of producing large 3D amorphous structures with complex geometry from BMGs [169]. Here, 100 μm-thick amorphous foils are laser welded in a layer-by-layer assembly to produce a 3D amorphous structure. Importantly, the printed structures display similar or better degree of amorphisation as the input thin foils. Key processing parameters include the control over heating and cooling rates. 

Laser power is another critical process parameter that affects amorphisation. When the Cu_66.5_Zr_33.5_ (at %) metallic glass layers were deposited using laser melting additive manufacturing technique [170], the resultant Cu-based metallic glass micro-wires were ~150 µm in diameter. Increasing the power of the laser beam increased the mean width of formed layer, from 166 to 356 µm, explained by a change in the wettability. Decreasing the power of the laser resulted in MBG structures with higher micro-hardness.

### 6.2. Shape-Memory Alloys

Shape-memory alloys are a class of biomaterials which are capable of undergoing reversible phase transitions as a result of temperature, pressure, or other stress-related effects, such as super elasticity [171]. These have unique mechanical and functional properties which are known as ‘the shape memory effect’ and their pseudo-elasticity [172]. Pseudo-elasticity explains the ability of a material to recover to its original shape after a mechanical load has formed large deformations. The shape memory effect describes the ability of a material to be plastically deformed below the transition temperature [173]. This material will then recover to its original shape once the temperature has increased. Shape memory alloys are characterised by two solid phases. The first solid type is the austenitic parent phase which is stable at high temperatures and has high symmetry [174]. The second solid type is the martensitic phase which is stable at low temperatures and has low symmetry. In regards to the application of this alloy, it is important to attain the desired mechanical characteristics, shape memory and pseudo-elastic behaviour at temperatures that suit the living systems [175]. 

Nitinol is a Ti-based thermos elastic biomaterial with 50% atomic Ni content. Ti-based materials have been the most promising shape memory alloys, which is why Nitinol has become so popular in the medical field [176]. Nitinol has mechanical stability, lower stiffness, and thermo-elasticity and biodegradation corrosion resistance. With these promising properties, Nitinol is a suitable replacement for stainless steel implants. Other applications for Nitinol according to its shape memory alloy characteristics include wires, palatal arches, intraspinal implants, intramedullary nails, staples, and devices for correcting scoliosis, spinal vertebrae spacer, self-expanding vascular stents and many more [177]. However, any use of Nitinol within the body hinges on the ability to control the release of Ni, which is typically achieved though surface modification in order to preserve bulk shape recovery properties of the alloy. Strategies typically used to modify the surface properties of Nitinol and thus control corrosion include deposition of thin film coatings, chemical etching, electro- and mechanical polishing, annealing, ion beam treatment, autoclaving, ion implantation, laser melting and others [178]. The depth of the modification also varies from nm to µm, with thicker coatings potentially providing better corrosion protection under strain. Yet, in very small implants, such as vascular stents, thinker coatings may also interfere with shape recovery ability of the implant. Presence of the Ni-enriched sub-layer, level of surface oxide hydration and surface charge, catalytic activity, conductivity and electronic structure of surface oxides, presence of structural defects and other surface and bulk characteristics may also be important factors that affect Ni ion leaching. 

3D printed porous nitinol skeletal fixation devices have the potential to provide sufficient stiffness for fixation during a 6–9 month healing period, and flexibility to restore normal stress distribution once the bone is fully remodeled [179]. It has been shown that when Ti-6Al-4V fixtures are used to repair segmental mandibulectomy defects, stress shielding may lead to the failure of the bone to remodel correctly, with the fixation device required to continue to carry most of the load. Under these circumstances, the implant may eventually break. For these cases, stiffness-matched nitinol fixation devices more adequately recreate normal stress-strain trajectories in a regenerated mandibular defect compared to Ti-6Al-4V, providing better chewing, speaking, swallowing, and breathing patient outcomes.

## 7. Conclusions

In this review, the currently used biomaterials have been discussed with possible drawbacks and suggestions to improve. It is the new or modified materials that can address these challenges currently faced by existing implants, and offer biomaterials that not only minimize the likelihood of medical complications but possibly offer more realistic, aesthetically pleasing outcomes for the patients. Materials engineering can add significant value to the future biomaterials and the next generation implants can be manufactured using nanomaterials which can serve multiple purposes.

3D printing holds great promise for processing of patient-specific metallic implants to create complex architectures with controlled internal nano-, micro-, and macro-scaled features and personalised external geometry. However, at present, there are a number of issues that need to be addressed for these sophisticated implants to be used clinically. These include advances in visualisation and modelling techniques. Indeed, even though CT scans are produced in very thin slices, the imaging modality can only generate the accumulation of the multiple slices, becoming a source of error. While this error may not be significant for macroscale features, the impact at micro- and nano-scale can be significant. Furthermore, the resolution of the assembly methods also needs to improve for faithful recreation of modelled architectures. Once these obstacles are overcome, 3D printing of metallic implants can develop into a mature modality in personalised medicine.

## Figures and Tables

**Table 1:** A comparison of mechanical properties of metallic implants with bone tissue [102,103].

Tissue/Material	Young’s Modulus (GPa)	Yield Strength (MPa)	Compression Strength (MPa)	Tensile Strength (MPa)
Cortical bone	7–30		100–230	164–240
Cancellous bone	0.01–3.0		2–12	
Ti_6_Al_4_V (casted)	114	760–880		895–930
Ti_6_Al_4_V (wrought)	114	827–1103	896–1172	860–965
Stainless steel 316L	193	170–310	480–620	540–1000
CoCrMo Alloy	240	500–1500		900–1540
Mg (99.9%, casted)	41	21	40	87
Mg (99.9%, wrought)	41	100	100–140	180
